# Quality control parameters on a large dataset of regionally dissected human control brains for whole genome expression studies

**DOI:** 10.1111/j.1471-4159.2011.07432.x

**Published:** 2011-10

**Authors:** Daniah Trabzuni, Mina Ryten, Robert Walker, Colin Smith, Sabaena Imran, Adaikalavan Ramasamy, Michael E Weale, John Hardy

**Affiliations:** * Reta Lilla Weston Laboratories and Departments of Molecular Neuroscience, UCL Institute of NeurologyLondon, UK; † MRC Sudden Death Brain Bank Project, Department of Neuropathology, University of EdinburghEdinburgh, , UK; ‡ Department of Medical & Molecular Genetics, King’s College London, Guy’s HospitalLondon, UK

**Keywords:** brain pH, microarray validation, post-mortem human brain, QTL, RIN, RNA

## Abstract

We are building an open-access database of regional human brain expression designed to allow the genome-wide assessment of genetic variability on expression. Array and RNA sequencing technologies make assessment of genome-wide expression possible. Human brain tissue is a challenging source for this work because it can only be obtained several and variable hours post-mortem and after varying agonal states. These variables alter RNA integrity in a complex manner. In this report, we assess the effect of post-mortem delay, agonal state and age on gene expression, and the utility of pH and RNA integrity number as predictors of gene expression as measured on 1266 Affymetrix Exon Arrays. We assessed the accuracy of the array data using QuantiGene, as an independent non-PCR-based method. These quality control parameters will allow database users to assess data accuracy. We report that within the parameters of this study post-mortem delay, agonal state and age have little impact on array quality, array data are robust to variable RNA integrity, and brain pH has only a small effect on array performance. QuantiGene gave very similar expression profiles as array data. This study is the first step in our initiative to make human, regional brain expression freely available.

Microarray analysis and RNA sequencing of the transciptome in post-mortem human brain tissue is a vital tool in investigating the complex genetic mechanisms involved in neurodegenerative and psychiatric disorders (Gilbert *et al.* 1981; Glanzer *et al.* 2004; Myers *et al.* 2007). However, there are many variables which influence the RNA integrity in post-mortem human brain tissues which need to be accounted for such data to be highly reliable (Sajdel-Sulkowska *et al.* 1988; Burke *et al.* 1991; Glasel 1995; Imbeaud *et al.* 2005; Schroeder *et al.* 2006; Birdsill *et al.* 2010; Durrenberger *et al.* 2010).

It is important to have a reliable and stable method to assess the quality of RNA samples generated from precious heterogeneous tissues, especially from small anatomical regions, such as the substantia nigra and hypothalamus. The most widespread measure for estimating the integrity of RNA samples at present is the RNA Integrity Number (RIN) as calculated by the Agilent 2100 Bioanalyzer for electrophoresis (Agilent Technologies UK Ltd, Edinburgh, UK). The RIN ranges from undetectable to ten, with undetectable being completely degraded and 10 being the most intact RNA. The calculation of RIN value is largely based on ribosomal RNA separation although this measure has been shown to be inconsistent (Imbeaud *et al.* 2005; Schroeder *et al.* 2006; Sherwood *et al.* 2011).

We are building a publicly accessible database of regional human brain expression, the UK Human Brain Expression Consortium, to allow the assessment of the genetic variability in gene expression (expression quantitative trait loci, eQTLs) and splicing (splicing quantitative trait loci, sQTL) as well as detailed genome-wide expression analysis (Hardy *et al.* 2009). To that end, we are collecting a large series of control human brain tissues (originating from ∼130 individuals) in which we are dissecting 13 different CNS areas: prefrontal cortex Brodmann areas 9 and 46, parietal cortex Brodmann areas 3,1, and 2, occipital cortex (OCTX) Brodmann areas 17, temporal cortex Brodmann areas 21,41 and 42, central white matter (WHMT) below Brodmann areas 39 and 40, hippocampus, thalamus, hypothalamus, putamen (PUTM), cerebellum (CRBL), substania nigra, medulla and spinal cord. From each individual brain, we isolated DNA for whole genome genotyping analysis and from each region we isolated RNA for whole transcriptome exon array analysis. This resulted in a total of 1266 RNA samples analysed on Affymetrix Exon arrays and represents by far the largest single CNS expression dataset at present. For this quality control study, we focused on analysing the factors that affected the reliability of the RNA samples.

In this study, we assess: (i) the effects of brain bank, age, gender, cause of death, region, post-mortem delay and brain pH on RIN-based RNA quality, and, (ii) the effects of RNA quality on the performance quality of the array experiment, which was measured by a reliable and widely used parameter, present call (%P). %P is the percentage of probe sets with signal detection above background noise. We examine the effects of RNA quality on the cDNA preparation and cRNA production as part of the quality control of the array experiment, and finally we confirm the reproducibility of array data using QuantiGene (QG), a novel, PCR-independent platform (Canales *et al.* 2006; Arikawa *et al.* 2008; Hall *et al.* 2011).

## Materials and methods

### Human post-mortem brain tissue collection and dissection

Brain tissues originating from 101 control Caucasian individuals were collected by the Medical Research Council (MRC) Sudden Death Brain and Tissue Bank (Edinburgh, UK; Millar *et al.* 2007). The bodies were stored refrigerated and were brought up to the PM suite just prior to the start of the autopsy. Each post-mortem brain dissection was carried out in the same way. The whole brain was removed within 15 min of the body as fresh tissue. The brain was immediately cut into coronal slices and the various anatomical regions of interest were immediately sampled. Furthermore, the samples once removed from the coronal slices were placed in sealed containers which in turn were placed on cool blocks (chilled to −20C) and stored within an insulated box. The samples were dissected into various size pieces approximately 250–500 mg and were placed in tubes which were immediately snap frozen in liquid nitrogen. The post-mortem interval (PMI) was calculated from the time of death to the time of removal of the brain from the skull. The total number of samples processed from this source was 1842 as some CNS regions were not available. In all cases, control status was confirmed by histology performed on sections prepared from paraffin embedded brain blocks and the diagnosis was determined by a consultant neuropathologist. Detailed phenotypic information is described in Table 1.

**Table 1:** Demographics of the samples studied. Values (range, mean) for variables in the cohort from both MRC-UK and SHRI-USA data set separately and joined. Of note, however, because of the different practices of the two tissue resources, there is no overlap in the PMIs between the MRC-UK (long PMI) and SHRI-USA (short PMI)

		Sex		Age/year		Brain pH		PMI (h)		RIN no.	
						
Brain bank	Individuals	Male	Female	Range	Mean	Range	Mean	Range	Mean	Range	Mean
MRC-UK	101	78	23	16–83	50.4	5.42–6.31	6.3	28–114	52.2	1–8.5	4
SHRI-USA	36	24	12	53–102	80	NA	NA	1–5.5	2.6	1–8	3.6
UK + USA	137	102	35	16–102	59	5.42–6.31	6.3	1–114	43.7	1–8.5	3.85

An additional 36 brains originating from neuropathologically confirmed control Caucasian individuals were collected by the Sun Health Research Institute (SHRI) an affiliate of Sun Health Corporation, USA (Beach *et al.* 2008). In this case, whole brains were removed as fresh tissue at autopsy and brain coronal slices were frozen. Anatomical regions of interest were sampled from brain coronal slices on dry ice. The time interval from the removal of the brain at the mortuary to the completion of the dissection and placement of samples within the storage freezer ranged from 2.5 to 4 h. The parietal cortex, hypothalamus and spinal cord regions were not available for these samples. The total number of samples processed was 476 as again, some regions were not available from some brains. Detailed phenotypic information is described in Table 1.

All samples from both sites had fully informed consent for retrieval and were authorised for ethically approved scientific investigation (Research Ethics Committee number 10/H0716/3).

### Post-mortem determination of brain pH

Brain pH from the MRC-UK samples was recorded from multiple regions using a Hanna HI8424 hand-held pH meter with a glass bodied electrode (Fisher Scientific, Loughborough, UK). Consistent with previous studies, the pH did not vary between different brain regions (Stan *et al.* 2006; Monoranu *et al.* 2009). We therefore used a single pH value that measured from the lateral ventricle. The electrode was inserted into the lateral ventricle after the brain was coronally cut behind the mamillary bodies. The pH is influenced by that of the tissue comprising the wall of the lateral ventricle and to a lesser extent by the pH of the remaining CSF fluid within the lateral ventricle. The pH was not measured in the SHRI-USA samples.

### RNA extraction

Total RNA was isolated from human post-mortem brain tissues based on the single-step method of RNA isolation (Chomczynski and Sacchi 1987) using the miRNeasy 96 kit (Qiagen, Crawley, UK). Brain tissues (50–100 mg) were collected and weighed in RNase-free 96-well plates. A minimum of one extraction was performed from each tissue sample. All steps were performed on dry ice prior to the addition of the QIAzol® Reagent. All samples were homogenised in 4°C using the TissueLyser II (Retsch, Castleford, UK) for 4–5 min at 30 Hz in 800 μL of QIAzol with the addition of two 3-mm stainless steel beads. In this step, the solution was homogenised until no large pieces remained.

The homogenised tissue samples were incubated at room temperature (15–25°C) for 5 min. After incubation, 160 μL of chloroform (CHCl_3_) (Sigma-Aldrich, Gillingham, UK) was added, the plates were mixed vigorously using the TissueLyser II for 30 s at 30 Hz. Plates were incubated at room temperature (15–25°C) for 7–10 min. The plates were centrifuged at 6000 *g* for 45 min at 4°C.

The aqueous phase (upper layer, with approximately 60% of the total volume after the QIAzol was added) was transferred to fresh RNase-free 96-well plates. RNA was precipitated by adding 800 μL of 100% ethanol to the aqueous phase (1.5 volume of the aqueous phase), followed by mixing the samples. The samples were then transferred into the RNeasy 96-well plates to allow the RNA in the solution to bind to the membrane by centrifugation.

The plates were centrifuged at 5600 *g* for 2 min at 21°C, and the supernatant was discarded. Samples were washed to remove salt traces and impurities by adding 600 μL of washing buffer and centrifuging plates for 2 min. Washes were performed 3–4 times. Finally, total RNA was eluted in 65 μL of pre-heated RNase-free water (50°C).

The concentration and purity of each RNA sample was assessed using the NanoDrop ND-1000 Spectrophotometer V3.3.0. The concentration of each sample was calculated, together with the ratio of absorbance at 260 nm/280 nm and 260 nm/230 nm.

After purification all RNA samples were applied to a RNA 6000 Nano-LabChip and analysed using the Agilent 2100 Bioanalyzer (Agilent Technologies UK Ltd) to obtain the RIN values. RINs and total RNA electropherograms were calculated by the 2100 Expert Software (Agilent Technologies UK Ltd). Further assessment of the RIN was performed by checking each electropherogram visually.

### Expression profiling using Affymetrix GeneChip® Human Exon 1.0 ST Arrays

Expression profiling on the Affymetrix GeneChip® Human Exon 1.0 ST Arrays (Affymetrix, High Wycombe, UK) was performed at AROS Applied Biotechnology AS company laboratories (http://www.arosab.com/).

Total RNA (200 ng) was used as starting material for the cDNA preparation. First and second strand cDNA synthesis, the *in vitro* transcription reaction to generate cRNA and the second round of cDNA synthesis was performed using the Ambion® WT Expression Kit according to the manufacturer’s instructions. Biotin labelling was performed using the Terminal Labeling Kit (Affymetrix) according to the manufacturer’s instructions. Following the *in vitro* transcription reaction, the unincorporated nucleotides were removed using RNeasy columns (Qiagen).

Prior to hybridisation, the fragmented cDNA was heated to 95°C for 5 min and subsequently to 45°C for 5 min before loading onto the Affymetrix Human Exon 1.0 ST array cartridge. The array cartridge was then incubated for 16 h at 45°C at constant rotation (60 rpm). The washing and staining procedure was performed in the Affymetrix Fluidics Station 450. The array was exposed to 10 washes in 6× SSPE-T at 250°C followed by four washes in 0.5× SSPE-T at 50°C. The biotinylated cRNA was stained with a streptavidin-phycoerythrin conjugate, final concentration 2 mg/mL (Affymetrix) in 6× saline sodium phosphate EDTA buffer with 0.01% Tween-20 (SSPE-T) for 30 min at 25°C followed by 10 washes in 6× SSPE-T at 25°C. This was followed by an antibody amplification step using normal goat IgG as blocking reagent, final concentration 0.1 mg/mL (Affymetrix) and biotinylated anti-streptavidin antibody (goat), final concentration 3 mg/mL (Affymetrix). This was followed by a staining step with a streptavidin–phycoerythrin conjugate, final concentration 2 mg/mL (Affymetrix, UK) in 6× SSPE-T for 30 min at 25°C and 10 washes in 6× SSPE-T at 25°C. The arrays were scanned at 560 nm using a confocal laser-scanning microscope (GeneChip® Scanner 3000 7G).

### Array quality control

The Expression Console™ (EC) software version 1.1 (Affymetrix) was used to evaluate the performance quality of the arrays including the labelling, hybridisation, scanning and background signals by Probe Set summarisation and CHP file generation using Robust Multichip Analysis. The quality assessment was performed by generating different parameters for all the probesets analysed by EC; %P is the main parameter that is used for the array quality in this study.

In addition, cDNA and cRNA Agilent 2100 Bioanalyzer profiles were generated for samples with wide range of RIN numbers (RINs from 2 to7) to assess the cDNA preparation and cRNA production nucleotide lengths from RNA samples with different levels of degradation.

### Exon array data analysis

All arrays were pre-processed using Robust Multichip Analysis quantile normalisation with GC background correction in Partek’s Genomics Suite v6.6 (Partek Inc., St. Louis, USA) (Lockstone 2011). After re-mapping the Affymetrix probe sets onto human Ref Seq build 19 (GRCh37) as documented in the most recent Netaffx annotation file (HuEx-1_0-st-v2 Probeset Annotations, Release 31), we restricted analysis to 308,717 probe sets that had a gene annotation and contained at least three probes with unique hybridisation. The gene-level expression was calculated for 27 000 genes by the median of probe sets corresponding to each gene.

### Array validation using direct RNA quantification with branched DNA, QuantiGene® 2.0 Assay

CRBL, OCTX, PUTM and WHMT samples from 12 individuals were analysed using the QG platform for validation of exon array results. We focused on three target genes for validation, leucine-rich repeat kinase 2 (LRRK2), sodium channel, voltage-gated, type VIII, alpha subunit (SCN8A), and microtubule-associated protein tau (MAPT). We selected ribosomal protein, large, P0 and ubiquitin C as housekeeping genes to normalise the target genes as they showed relatively low variability in expression levels (i.e. low coefficient of variation) in all brain regions in our dataset. The approach to the selection of reference genes is explained in previous studies (de Jonge *et al.* 2007; Coulson *et al.* 2008).In addition, a recent study confirms the efficiency of using this approach in selecting housekeeping genes to normalise in different tissues (Chervoneva *et al.* 2010). A summary of the QG probes used for analysis of all five genes is provided in Table 2.

**Table 2:** QuantiGene probes used to perform array validation

Gene	Catalogue no.	Designed to hybridise to
LRRK2	83322 SA-26988	Human LRRK2
SCN8A	83324 SA-17320	Human SCN8A transcript variants 1 and 2
MAPT	81849 SA-15486	Human MAPT, all six variants
UBC	80041 SA-10061	Human UBC
RPLP0	81152 SA-11148	Human RPLP0 transcript variants 1 and 2

QuantiGene 2.0 Reagent System was used and the protocol in the QuantiGene 2.0 Reagent System User Manual was followed with the exception of the substrate step. Lumigen® Lumi-Phos® Plus and 10% lithium lauryl sulfate was used instead of Lumigen® APS-5 substrate. A Biotek ELx 405 select plate washer was used for all of the wash steps in the assay. The QG 2.0 plates were then read on a Molecular Devices LMax luminometer with the plate incubator set to 45°C to maintain the temperature of the Lumigen® Lumi-Phos® Plus substrate. In total, 13 QG 2.0 plates were run to cover all target genes and the house keeping genes. Each house keeping gene ribosomal protein, large, P0 and ubiquitin C was loaded in duplicates at 12.5 ng/well. In addition, target genes (LRRK2, SCN8A and MAPT) were loaded in duplicates at 75 ng/well.

### Statistical analysis

Linear mixed regression analyses were performed to investigate the relationship between age, gender, cause of death, region, PMI, pH, RIN and present call (%P). Statistical analyses were conducted using Partek® Genomics Suite™ version 6.6 and PASW statistic version 18 software. We assessed explanatory power in a forwards stepwise manner, by examining the increase in variation explained when a new covariate or set of covariates were added to the existing model, together with a *p*-value for that increase.

## Results

**Fig. 1:**
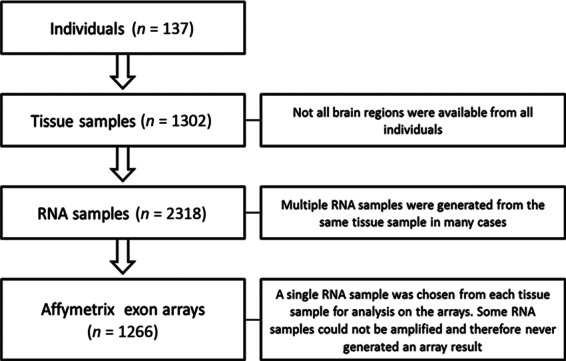
Description of the data used in the analysis.

This study involved the analysis of brain tissue originating from 137 individuals. Since we were not able to obtain all 13 brain regions of interest from each individual, a total of 1302 tissue blocks were available. In the majority of cases (870 of 1302 tissue blocks) multiple RNA extractions were performed, resulting in 2318 RNA samples in total. Each of these RNA samples was assessed for RNA integrity as measured by RIN. A single RNA extraction from each tissue sample was selected on the basis of Agilent 2100 Bioanalyzer profile for downstream analysis using the Affymetrix Exon arrays (Fig. 1).

### The effect of age, gender, cause of death, region, PMI and brain pH on RIN-based RNA quality

We assessed the dependence of RIN values on the other covariates available to us. Our focus was on the explanatory power of our available covariates on the RIN for each RNA extraction (2318 RNA samples). In this study, we assessed 2318 RNA samples. The calculated RIN ranged from 1 to 8.5 with a mean of 3.85 (Table 1). Forty-three per cent of our samples had RIN values of < 3. We found that 33% of the variation in RIN was explained by differences among tissue blocks (adjusted *R*^2^ measure), which set an upper limit for the explanatory power of our covariates, which all act at a between-tissue-sample level. Sixteen per cent of the variation in RIN was explained by individual-level differences.

Outside of pH and PMI, which were analysed separately, the most important covariates were brain region (explained 9.2% of the variation in RIN number, *p* = 1.7 × 10^−42^ ), age (explained an additional 1.1%, *p* = 4.1 × 10^−05^ ) and cause of death (explained an additional 1.9%, *p* = 0.022). Brain bank and gender together explained an additional 0.4% of the variation.

pH and PMI were investigated separately, because pH was not measured in the SHRI-USA dataset and because the range of PMIs from the two brain banks did not overlap.

**Fig. 2:**
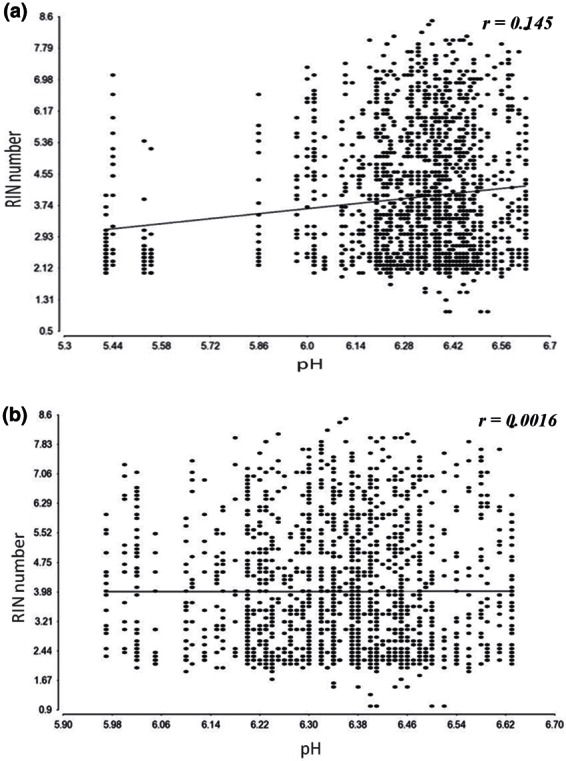
(a) Scatter plot for total RNA samples with linear regression line of RIN numbers for pH. Plot shows the effect of pH on RIN number for RNA samples isolated from 13 region of control brain tissue. Test *p*-values is test *p*-value = 1.0 × 10^−4^, *r*-value = 0.145. Including the low pH values of < 5.9. (b) Scatter plot for total RNA samples with linear regression line of RIN numbers for pH. Plot shows the effect of pH on RIN number for RNA samples isolated from 13 region of control brain tissue. No significant correlation was obtained with *r*-value = 0.0016 and *p*-value = 0.82. Samples with low pH value of < 5.9 were excluded.

pH explained 2.1% of the variation in the RIN number from the MRC-UK brain bank (*p* = 1.0 × 10^−4^ ) (Fig. 2a).We note that six individuals had very low pH < 5.90 and when excluded, the correlation was no longer significant (Fig. 2b).

The effect of PMI on RIN differed between the MRC-UK and SHRI-USA datasets. There was no significant correlation between PMI and RIN in the MRC-UK dataset, which had PMIs ranging from 28 to 114 h. In contrast, PMI explained 8.4% of the variation in RIN in the SHRI-USA dataset (*p* = 0.0053), which had PMIs ranging from 1 to 5.5 h. Longer PMI was associated with a higher RIN in this data set, a counterintuitive result which might represent confounding with some unmeasured variables in the study.

### The effects of age, gender, cause of death, region, PMI, brain pH and RIN-based RNA quality on array performance (%P, cDNA and cRNA profile)

We assessed the dependence of RNA array performance quality on RIN-based RNA quality and the other covariates available to us. A systematic quality control check of the arrays was performed using Expression Console™ software. This software produces a number of array quality measures. The most reliable and widely used parameter is the present call (%P) (Tomita *et al.* 2004; Weis *et al.* 2007). The percent present call is the percentage of probe sets with signal detection above background probe level *p* -value of ≤ 0.01. The range of %P in this study was 1.7–77.3% (mean of 61.4%). Thrity-six per cent of the variation in %P was explained by individual-level differences (adjusted *R*^2^ measure).

**Fig. 3:**
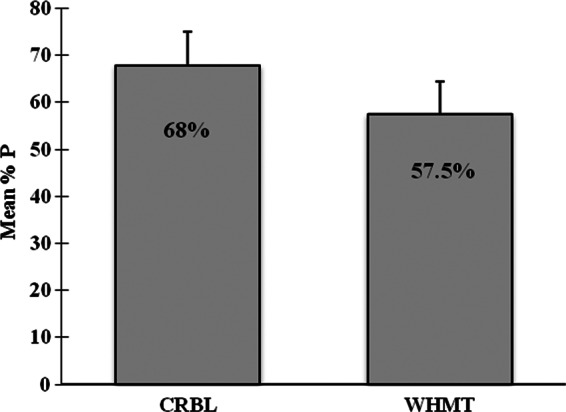
Bar chart to show variation in %P by brain region (CRBL, WHMT).This graph shows the different performance of samples from specific brain regions on the array. These results are highly significant *p*-values (1.3 × 10^−24^ ). The heights of the bars represent the mean. The error bars represent the SEM.

Outside of pH and PMI, which for reasons described previously were analysed separately, the most important covariates were brain region (explained 12.4% of the variation in %P, *p* = 3.6 × 10^−53^ ), followed by brain bank (explained an additional 4.7%, *p* = 9.6 × 10^−6^ ), and then RIN (explained an additional 2.7%, *p* = 9.1 × 10^−05^ ). Age, gender, and cause of death together explained an additional 2.2% of the variation. The effect of brain region on %P was most obvious when comparing CRBL and WHMT. CRBL showed highest %P (mean = 68%) whereas WHMT showed the lowest %P (mean = 57.5%) (Fig. 3).

**Fig. 4:**
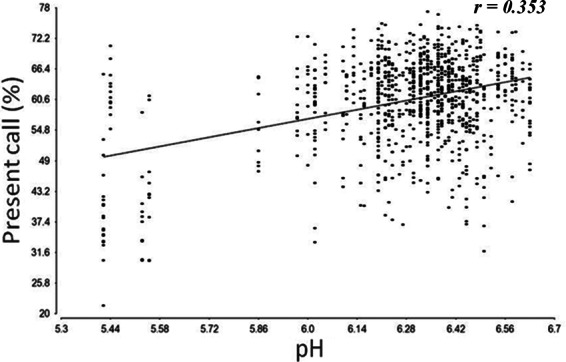
Scatter plot for total RNA samples with linear regression line of Present call (%P) for pH. Scatter plot shows that pH significantly explains 12.0% of the variation in %P (*p* = 2.3 × 10^−9^, *r* = 0.353), including samples with low pH values. Low pH values are driving the regression analysis.

pH explained 12.0% of the variation in %P from the MRC-UK brain bank (*p* = 2.3 × 10^−9^ ). (Fig. 4). However, as with RIN, this correlation was highly dependent on the six individuals with low pH < 5.90. When these samples were excluded from the analysis no significant correlation was obtained.

Finally, no significant correlation was found between PMI and %P, either for the MRC-UK dataset or the SHRI-USA dataset.

Moreover, no difference was observed in the expected nucleotide lengths following cDNA preparation (∼200–400 nt) and cRNA production (ranges from 200 to 2000 nt) between different samples with different RIN values (as shown by Agilent Bioanalyser profiles).

### The reproducibility of array data using QuantiGene, a PCR-independent platform to confirm expression results

QuantiGene is a technique for mRNA expression quantification. Its strength is that it is a non-PCR based technique that is therefore not subject to the systematic biases that PCR could create when applied to degraded RNA. This technique has the potential to be used in place of qRT-PCR (Canales *et al.* 2006; Hall *et al.* 2011).

**Fig. 5:**
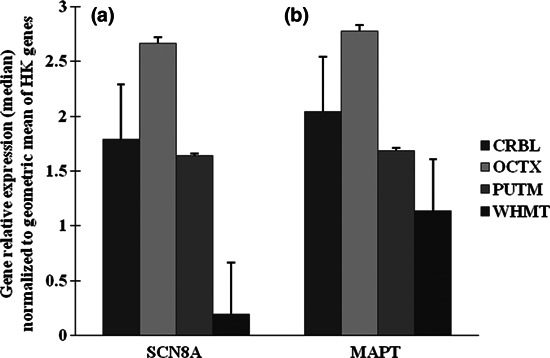
QuantiGene validation of microarray expression data. (a) SCN8A expression level between different regions. The graph shows high expression in OCTX compare with other regions. It is clear that this gene in mostly not expressed in WHMT region (expression level close to zero) also it presenting large error bar. (b) MAPT, showing higher expression in OCTX compare with other brain regions. These results confirm the array data with significant *p*-values of < 0.01. The expression level is presenting the mean and the error bars is SEM.

**Fig. 6:**
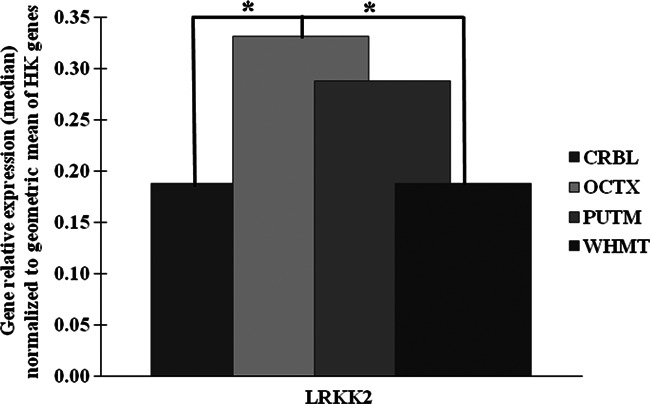
QuantiGene validation of microarray expression data for LRRK2 expression level in different regions. The graph shows higher expression in OCTX compare with other regions. Wilcoxon-signed rank test was performed and these results confirm the difference between regions in array data is significant. The expression level is presenting the median and the stars indicating the significant difference in expression with *p*-values of < 0.01. In this case, as LRRK2 expression level was very low, it was unreliable to use SEM and Wilcoxon’s test was performed using the median values.

We validated the exon array results in a subset of 12 individuals by comparing the normalised mRNA expression level for three genes (LRRK2, SCN8A and MAPT), in four brain regions (CRBL, OCTX, PUTM and WHMT). We observed a similar regional pattern of expression across the two platforms for both high (SCN8A and MAPT) (Fig. 5) and low expression transcripts in brain (LRRK2) (Fig. 6).

Furthermore, there was an excellent correlation in fold change (*r*^2^ = 0.855) at both the gene and exon expression levels and signal intensity values (*r*^2^ = 0.91) in the four brain regions we studied.

## Discussion

To our knowledge, the UK Human Brain Expression Consortium data set is the largest control brain microarray data set generated to date. It is based on the analysis of tissue samples from 13 different CNS regions originating from 137 individuals and containing 2318 processed samples. The main goal of this project is to build a large reference database for eQTL and sQTL analysis.

This study showed considerable variation in RIN values among RNA samples. Sixty-seven per cent of the variation resides in differences among extractions from the same tissue blocks and most of the remaining variation is unexplained by the available covariate information. We found that pH is the most important post-mortem factor influencing RIN-based RNA integrity, a result consistent with previous studies (Hardy *et al.* 1985; Mexal *et al.* 2006; Chevyreva *et al.* 2008; Monoranu *et al.* 2009; Durrenberger *et al.* 2010). Samples with very low pH values (ranging from 5.42 to 5.90) were responsible for the positive correlation seen between pH and %P (and also RIN). However, when these low pH samples were removed from the analysis we no longer observed any significant correlation. This may in part explain contradictory observations regarding the effect of pH on RNA integrity and sample performance on arrays (Hardy *et al.* 1985; Monoranu *et al.* 2009; Birdsill *et al.* 2010) and confirms the findings of a recent, but smaller study on control brain tissue (Tomita *et al.* 2004; Sherwood *et al.* 2011).

We found our array-based expression data to be reliably validated by the QuantiGene PCR-independent method, when tested on two high expression genes (MAPT and SCN8A) and one low expression gene (LRRK2). However, the performance quality of the array, as defined by %P, was not profoundly affected by age, gender, region, PMI, RIN and cause of death. This confirms findings from previous studies on much smaller sample sizes (Tomita *et al.* 2004; Birdsill *et al.* 2010; Durrenberger *et al.* 2010). We found that only 2.7% of the variation in %P was explained by RIN. Indeed, 80 RNA samples with undetectable RINs performed well on the arrays with %P values ranging from 45 to 76%. Thus, we found RIN to be a poor predictor of array quality performance even at the low end of the RIN scale. Furthermore, the latter was confirmed since the cDNA and cRNA length synthesis was not affected by the wide range of RIN values (from 2 to 7) in our array experiments. The robust performance of the Affymetrix Exon arrays in the face of degraded RNA may be due to recent changes to the RNA amplification process. In keeping with the manufacturer’s instructions, this was performed using the Ambion® WT Expression kit, which uses both non-polyA and polyA-based mRNA priming for first strand cDNA synthesis. This meant that RNA amplification did not require an intact polyA tail. In addition, increasing the quantity of the starting material of RNA from 500 to 750 ng improved the array performance.

Through the analysis of this observational study, we experienced different limitations. For example, we had expected that cause of death would greatly influence both RIN-based RNA quality and %P-based array quality, but cause of death only explained 1.9% of variation in RIN and we did not find any significant relationship with %P. It may be that cause of death is an imperfect reflection of the true medical and drug treatment history of the individual, and that access to that history, were it available, would reveal other factors of greater relevance. Likewise, in the range of 28–114 h PMI did not affect on either RIN or %P, nor could we see a loss of RIN-based RNA quality over the 1–5 h range. It remains possible that there may be selective loss of RNA within an hour because the half-life of some mRNA species has been reported to be as short as 15 min, while others may be as long as 22 days depending on the tissue type and storage conditions (Ross 1995; Barrachina *et al.* 2006; Bahar *et al.* 2007; Beach *et al.* 2008; Vennemann and Koppelkamm 2010). This issue has been studied in detail for a range of PMIs by Harrison *et al.* 1995 and confirmed by a more recent study by Tomita *et al.* (2004). The same conclusion was made that PMI had a limited effect on mRNA (Harrison *et al.* 1995; Tomita *et al.* 2004).

Furthermore, there is a possibility that samples with the shortest PMIs (1–2.5 h) within the SHRI-USA sample set may originate from those individuals who suffered longer agonal states prior to death and agonal stress has been shown to affect gene expression differently in different brain regions (Li *et al.* 2007). Other factors may contribute to this such as intermittent edge effects, especially on small samples during dissecting and tissue handling procedures.

Finally, we note that our brain samples have been derived from only two sources: one was a rapid death brain bank with long post-mortem intervals and the other specialises in obtaining very short post-mortem intervals. Both brain banks had separately optimised their protocols to facilitate gene expression studies and this may limit the generalisability of these conclusions to tissue collected in other ways.

These results are important for several reasons. Firstly, they confirm the practical feasibility of using post-mortem control brain tissue to study the transcriptome of the human brain by array technology. Secondly, they show that microarrays can give reliable results over a wide range of RIN numbers (1–8.5) and pH measurements with drop off in array validity only being observed below brain pH 5.9. Thirdly, they show that the results from Affymetrix exon arrays are reproducible by other technologies, making it possible for database users to use the data generated with confidence.

Furthermore, this study is the first step of an ongoing multi-regional human brain expression project that has been established to build an open-access database of identified genome-wide genetic variability in relation with gene eQTLs and sQTL as well as for detailed expression analysis (Hardy *et al.* 2009). We hope this will move the field forward in our understanding of the underlying molecular mechanisms of complex neurological and psychiatric diseases, and will support the neuroscience community with a resource which will bring functional insights.

## Abbreviations

CRBL: cerebellum
eQTL: expression QTL
LRRK2: leucine-rich repeat kinase 2
MAPT: microtubule-associated protein tau
MRC: Medical Research Council
OCTX: occipital cortex
PMI: post-mortem interval
PUTM: putamen
QG: QuantiGene
RIN: RNA Integrity Number
SCN8A: sodium channel, voltage-gated, type VIII, alpha subunit
sQTL: splicing QTL
SHRI: Sun Health Research Institute
WHMT: white matter
